# ALT-to-Lymphocyte Ratio as a Predictor of Long-Term Mortality in Patients with Normal Liver Function Presenting Coronary Artery Disease after Undergoing PCI: A Retrospective Cohort Study

**DOI:** 10.1155/2020/4713591

**Published:** 2020-04-21

**Authors:** Ru-Jie Zheng, Qian-Qian Guo, Jun-Nan Tang, Xu-Ming Yang, Jian-Chao Zhang, Meng-Die Cheng, Feng-Hua Song, Zhi-Yu Liu, Kai Wang, Li-Zhu Jiang, Lei Fan, Xiao-Ting Yue, Yan Bai, Xin-Ya Dai, Zeng-Lei Zhang, Ying-Ying Zheng, Jin-Ying Zhang

**Affiliations:** ^1^Department of Cardiology, First Affiliated Hospital of Zhengzhou University, Zhengzhou 450052, China; ^2^Key Laboratory of Cardiac Injury and Repair of Henan Province, Zhengzhou, China; ^3^Department of Cardiology, The First Affiliated Hospital, College of Clinical Medicine of Henan University of Science and Technology, Luoyang 471003, China

## Abstract

**Background:**

Alanine aminotransferase (ALT) is referred as liver transaminase and predominantly expressed by hepatocytes. Previous evidences showed that high levels of ALT were reversely associated with short- and long-term outcomes in patients with myocardial infarction. Besides, low lymphocyte has been demonstrated to be significantly correlated with adverse clinical outcomes in coronary artery disease (CAD). However, evidences about the relationship between ALT-to-lymphocyte ratio (ALR) and outcomes in CAD patients with normal liver function are limited. The aim of this study was to assess the relationship between ALR and clinical outcomes in patients with CAD.

**Methods:**

This is a retrospective cohort study, and a total of 3561 patients were enrolled in Clinical Outcomes and Risk Factors of Patients with CAD after percutaneous coronary intervention (PCI), from January 2013 to December 2017. After excluding patients with liver dysfunction, we finally enrolled 2714 patients. These patients were divided into two groups according to ALR value: the lower group (ALR < 14.06, *n* = 1804) and the higher group (ALR ≥ 14.06, *n* = 910). The average follow-up time was 37.59 ± 22.24 months.

**Results:**

We found that there were significant differences between the two groups in the incidence of all-cause mortality (ACM) (*P* < 0.001) and cardiac mortality (CM) (*P*=0.010). Kaplan–Meier survival analysis suggested that CAD patients with higher ALR tended to have an increased accumulated risk of ACM and CM (log rank *P* < 0.001 and *P*=0.006, respectively). Multivariate Cox regression analysis showed that ALR was an independent predictor of ACM (hazard ratio (HR) = 2.017 (95% confidence interval (CI): 1.289–3.158), *P*=0.002) and CM (HR = 1.862 (95% CI: 1.047–3.313), *P*=0.034). We did not find significant difference in the incidence of major adverse cardiovascular events (MACEs) and major adverse cardiovascular and cerebrovascular events (MACCEs) between the two groups after adjustments of confounders.

**Conclusion:**

Our results indicate that ALR is an independent predictor of long-term adverse outcomes in CAD patients who underwent PCI.

## 1. Introduction

Although the management of coronary artery disease (CAD) has developed and improved dramatically during the past two decades, CAD is still a disease with increasing morbidity and mortality posing a major threat to human's health [1, 2]. The pathogenesis of CAD includes several mechanisms [3], such as lipid metabolism [4, 5], inflammatory response [6, 7], and activation of the coagulation and fibrinolysis system [8]. Alanine aminotransferase (ALT) is referred as liver transaminase and predominantly expressed by hepatocytes. Therefore, ALT represents a specific biomarker for hepatocellular injury. Previous evidences showed that high levels of ALT were reversely associated with short- and long-term outcomes in patients with myocardial infarction [9, 10]. Several other studies elucidated that elevated ALT was also associated with a higher prevalence of triple vessel CAD [11]. Besides, lymphocyte is vital element in smooth-muscle proliferation and intimal lesions [12, 13]. Low lymphocyte count has been demonstrated to be significantly correlated with adverse clinical outcomes in patients with CAD [14]. While much attention was paid to studies about ALT and lymphocyte, respectively, there was still a gap of our knowledge on the relationship between ALT-to-lymphocyte ratio (ALR) and clinical outcomes. As already mentioned above, all previous studies enrolled patients no matter the liver function is normal or abnormal. Our study excludes the patients with liver dysfunction and aims to investigate the relationship between ALR and clinical outcomes in patients with CAD.

## 2. Methods

### 2.1. Study Design and Population

In this study, 3561 CAD patients admitted in the Clinical Outcomes and Risk Factors of Patients with Coronary Heart Disease after PCI (CORFCHD-ZZ, identifier: ChiCTR1800019699) study were included. The CORFCHD-ZZ study is a large, single-center retrospective cohort study. These patients were angiography-proven CAD patients hospitalized at the First Affiliated Hospital of Zhengzhou University from 2013 to 2017 and received at least one stent via implantation. The exclusion criteria were patients who had dysfunction of the liver, serious heart failure, congenital disease, and serious dysfunction of the kidney. This study protocol complies with the Declaration of Helsinki and was approved by the ethics committee of the First Affiliated Hospital of Zhengzhou University. The ethics committee waived the need of obtaining informed consent from eligible patients, because of the retrospective design of this study.

**Figure 1:**
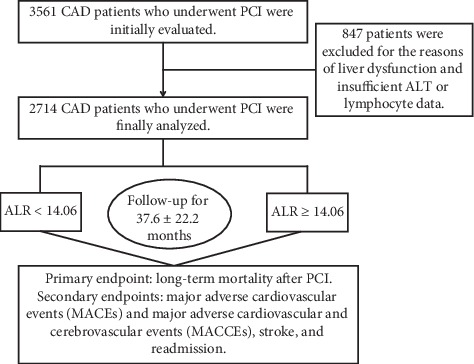
The flowchart of patients' enrollment.

To assess the correlation between the ALR and clinical outcomes in patients who had CAD and underwent PCI, 3561 patients were initially evaluated. 847 patients were excluded for the reason of liver dysfunction and insufficient ALT or lymphocyte data. Finally, 2714 patients were admitted in this study.  Figure 1 shows the flowchart of the inclusion and exclusion criteria used in the selection of participants.

### 2.2. Clinical and Demographic Characteristics Collection

The demographic data, laboratory data, and cardiovascular risk factors were collected and recorded for all study population. The cardiovascular risk factors include alcohol consumption, smoking status, history of hypertension, family history of CAD, and previously diagnosed diabetes.

Demographic data including height and weight were collected when the participants wore light clothing without shoes. According to the American Heart Association recommendations [15], hypertension was defined as the patient with blood pressure measurements ≥140/90 mmHg on at least three resting measurements on three mornings or receiving treatment of antihypertensive drugs. Patients with diabetes mellitus were defined as receiving treatment of glucose-lowering agents or a fasting plasma glucose ≥7.1 mmol/L or 2-hour post-load glucose ≥11.1 mmol/L [16]. The diagnostic criteria for hyperlipidemia were mainly obtained from the “Guideline of Chinese Adult Dyslipidemia Prevention and Treatment (2016)” [17]. The classifications of smoking status were current smokers, former smokers, and never-smokers. Current smokers were considered as persons regularly using tobacco in the last 6 months. Persons who ingest alcohol in the previous 6 months were considered alcohol users [18].

### 2.3. ALT Detection and Lymphocyte Count Detection

Peripheral blood samples were taken into vacutainer tubes at the time of admission, after 12 hours of fasting. The vacutainer tubes use sodium heparin as anticoagulant. Measurement of serum ALT activity and lymphocyte count were measured using a standard method in accordance to the central laboratory standard of the First Affiliated Hospital of Zhengzhou University. The normal concentration of serum ALT is <50 U/L for men and <40 U/L for women [19, 20]. ALR was calculated by dividing the serum ALT level with lymphocyte count.

### 2.4. Endpoints

The long-term mortality including all-cause mortality (ACM) and cardiac mortality (CM) was chosen as the primary endpoint. The secondary endpoints were readmission, major adverse cardiovascular events (MACEs) [21], defined as the combination of cardiac death, recurrent myocardial infarction, and target vessel reconstruction, and major adverse cardiac and cerebrovascular events (MACCEs) [22] defined as MACEs plus stroke. Stroke was defined as an acute neurologic deficit causing by cerebrovascular disease, containing haemorrhage, embolism, thrombosis, or aneurysm rupture, and lasting for >24 hours [22]. All events were determined by an adjudication committee which was blinded to the group of patients.

### 2.5. Follow-Up

In our study, we reviewed all medical records and contacted the patients or their families by telephone. All the patients were followed up at least 18 months. At each contact, the compliance of the drugs and adverse outcomes were assessed.

### 2.6. Statistical Analyses

All analyses were conducted using the SPSS 22.0 (SPSS, Chicago, Illinois). Continuous data were shown as the mean ± standard deviation. Categorical data were presented as the percentages and frequencies. The ALR was analyzed as a categorical variable divided into two groups on the basis of the ALR cut-off value of 14.06. The cut-off value (14.06) is according to the analysis of the ROC curve for the baseline ALT-to-lymphocyte ratio of the study population. The differences between normally distributed numerical variables were analyzed by a *t*-test, and nonnormally distributed variables were analyzed by the Mann–Whitney *U*-test or Kruskal–Wallis variance analysis as appropriate. Categorical variables were compared with Chi-square test. Cumulative incidence rate of long-term clinical outcomes was analyzed by using Kaplan–Meier analysis. The log-rank test was employed for comparing between two groups. Multivariable regression analysis was used to evaluate the predictive value of the ALR for outcomes during follow-up. Hazard ratios (HRs) and 95% confidence intervals (CIs) were calculated, and a two-sided *P* value <0.05 was considered statistically significant.

## 3. Results

### 3.1. Baseline Data

The patients were categorized into two groups according to the ALR value as described above: the lower ALR group (<14.06, *n* = 1804) and the higher ALR group (≥14.06, *n* = 910). As shown in Table 1, several variables, including age, gender, family history, diabetes mellitus, creatinine (Cr), total cholesterol (TC), triglyceride (TG), and low-density lipoprotein cholesterol (LDL-C), were significantly different between the two groups (all *P* values <0.05). We did not find any significant differences in respects of alcohol drinking, smoking, heart rate, hypertension, blood urea nitrogen (BUN), uric acid (UA), and high-density lipoprotein cholesterol (HDL-C) (all *P* values ≥0.05).

### 3.2. Clinical Outcomes

As shown in Table 1, for the primary endpoint, the incidence of ACM in the lower ALR group was 48 (2.7%), which in the higher ALR group was 50 (5.5%), and the difference was significant (*P* < 0.001); besides, the incidence of CM between these two groups also showed significant difference (1.7% *vs* 3.2%, *P*=0.010). For the secondary endpoints, we found that there were no significant differences in the incidence of MACEs (11.9% *vs* 11.1%, *P*=0.530), MACCEs (15.8% *vs* 14.6%, *P*=0.420), and stroke (4.4% *vs* 3.8%, *P*=0.513) between the two groups. Similarly, the incidences of cardiac insufficiency (16.0% *vs* 13.3%, *P*=0.061), bleeding (2.9% *vs* 2.7%, *P*=0.841), readmission (31.3% *vs* 27.7%, *P*=0.055), and secondary MI (3.2% *vs* 2.4%, *P*=0.278) did not show any significant differences.

Significant different variables (*P* < 0.05) which were shown in univariate models for each of the predictor variables entered into multivariate Cox regression analysis. To access the prognostic value of the ALR, we performed the multivariable analysis after adjusting for the traditional clinical prognostic factors, including age, gender, family history, diabetes mellitus, Cr, TC, TG, and LDL-C. We found that patients in the higher ALR group were at a higher accumulated risk for ACM and CM. During the long-term follow-up, compared with the lower ALR group, the risk for ACM and CM increased by 101.7% (HR = 2.017, 95% confidence interval (CI): 1.289–3.158, *P*=0.002), 86.2%(HR = 1.862, 95% CI: 1.047–3.313, *P*=0.034) in the higher ALR group, respectively (Tables 2 and 3).

**Figure 2:**
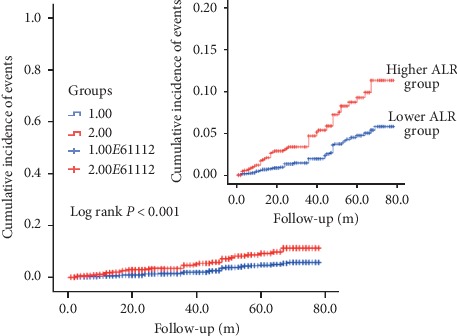
Cumulative Kaplan–Meier estimates of the time to the first adjudicated occurrence of all-cause mortality. The *X* axis represents the follow-up time, and the *Y* axis represents the cumulative incidence of ACM. The longest follow-up time is almost 80 months. The red line indicates the higher ALR, and the blue line indicates the lower ALR.

**Figure 3:**
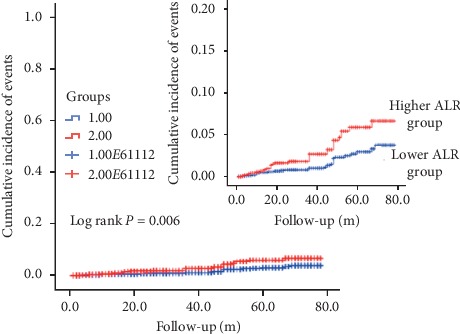
Cumulative Kaplan–Meier estimates of the time to the first adjudicated occurrence of cardiac mortality. The *X* axis represents the follow-up time, and the *Y* axis represents the cumulative incidence of CM. The longest follow-up time is almost 80 months. The red line indicates the higher ALR, and the blue line indicates the lower ALR.

In Figures 2 and 3, as Kaplan–Meier survival analysis revealed, patients underwent PCI with higher ALR level were apt to have an increasing accumulated risk for ACM and CM (log rank *P* < 0.001 and *P*=0.006, respectively).

## 4. Discussion

In this study, we demonstrated an increased ALR level was independently associated with adverse outcomes and found that ALR was a novel predictor of ACM and CM in patients who underwent PCI. To the best of our knowledge, this study is the first one to investigate the relationship between the ALR index and adverse outcomes in patients with CAD after PCI without abnormal liver function.

In recent years, a large number of novel biomarkers were found to detect the early damage of cardiomyocytes in clinical practice. These emerging biomarkers include microRNAs [23] and LncRNAs [24]. However, their performing detection methods were complicated and expensive. Hence, the predictive performance of these biomarkers on the outcomes was limited. More recently, several efficient biomarkers, including gamma-glutamyl transferase (GGT) [25, 26], GGT-to-platelet ratio (GPR) [27], mean platelet volume (MPV) [28, 29], red blood cell distribution width (RDW) [30, 31], and monocyte-to-HDL-C ratio [32], emerged. ALT, mainly derived from the liver, was considered as a marker of liver function. Studies have demonstrated that ALT was associated with an increased incidence of short- and long-term all-cause mortality in patients presenting STEMI who underwent PCI [10]. Similarly, Lofthus et al. [33] illustrated a strong relation between elevated ALT and poor clinical outcomes. In line, the isolated effect of ALT on 90-day mortality after cardiogenic shock [34] was demonstrated in several other studies. Moreover, other studies [35–37] had found an interesting fact that elevation of common marker, including ALT, results from congestion or ischemia. This finding could explain why our results indicated a relationship between ALR and CM. However, these studies did not exclude patients with alcohol abuse and dysfunction of liver. Abnormal liver function may have an influence on results. Our study excluded patients with abnormal liver function. Lymphocyte [7, 14, 38] was considered as a crucial role in the progression of smooth-muscle proliferation and intimal lesions. Lymphocyte involved in the regulatory pathway of the immune system modulates the inflammatory response [39]. Previous studies have demonstrated that low lymphocyte count was significantly correlated with adverse clinical outcomes in patients with CAD [14]. Of note, no attention was paid to the ALT-to-lymphocyte ratio, while several researches have confirmed the potential of ALT and lymphocyte on predicting mortality, leaving a major gap of knowledge. Fortunately, our study showed that the ratio of ALT to lymphocyte (ALR) is a novel, reliable, effective predictor for clinical outcomes of CAD.

There are several advantages of our study. One strength is the large sample size, and the large sample may make our results more reliable. The other strength is the long-term follow-up for all included patients.

## 5. Conclusion

The result of our study indicates that the ALR is an inexpensive, reliable, effective predictor for poor outcomes in patients presenting CAD who underwent PCI.

## Figures and Tables

**Table 1:** Characteristics of participants of the two groups and clinical outcomes.

Variables	ALR < 14.06	ALR ≥ 14.06	*χ2* or *t*	*P*
	*n* = 1804	*n* = 910		
Age, years	63.313 ± 10.571	64.245 ± 10.737	−2.157	0.031
Gender (male), *n* (%)	1163 (64.5)	673 (74.0)	24.882	<0.001
Family history, *n* (%)	354 (19.7)	148 (16.5)	4.067	0.044
Heart rate, beats/min	74.170 ± 21.841	74.440 ± 11.617	−0.339	0.734
Smoking, *n* (%)	535 (29.7)	280 (30.8)	0.357	0.55
Drinking, *n* (%)	288 (16.0)	150 (16.5)	0.12	0.729
Hypertension, *n* (%)	1044 (57.9)	493 (54.2)	3.364	0.067
Diabetes mellitus, *n* (%)	498 (27.6)	191 (21.0)	13.979	<0.001
BUN, mmol/L	5.833 ± 4.694	5.703 ± 4.453	0.685	0.493
Cr, mmol/L	71.608 ± 34.046	75.747 ± 46.709	−2.617	0.009
UA, mmol/L	297.273 ± 83.160	303.531 ± 88.172	−1.805	0.071
TG, mmol/L	1.713 ± 1.140	1.594 ± 1.128	2.528	0.012
TC, mmol/L	3.935 ± 1.013	3.753 ± 1.035	4.286	<0.001
HDL-C, mmol/L	1.043 ± 0.304	1.030 ± 0.298	1.024	0.306
LDL-C, mmol/L	2.421 ± 0.858	2.279 ± 0.814	4.043	<0.001
All-cause mortality, *n* (%)	48 (2.7)	50 (5.5)	13.956	<0.001
Cardiac mortality, *n* (%)	30 (1.7)	29 (3.2)	6.605	0.01
MACEs, *n* (%)	215 (11.9)	101 (11.1)	0.394	0.53
MACCEs, *n* (%)	285 (15.8)	133 (14.6)	0.65	0.42
Stroke, *n* (%)	79 (4.4)	35 (3.8)	0.427	0.513
Bleeding, *n* (%)	52 (2.9)	25 (2.7)	0.04	0.841
Readmission, *n* (%)	564 (31.3)	252 (27.7)	3.67	0.055
Cardiac insufficiency, *n* (%)	289 (16.0)	121 (13.3)	3.498	0.061
Secondary MI, *n* (%)	57 (3.2)	22 (2.4)	1.179	0.278

BUN, blood urea nitrogen; Cr, creatinine; UA, uric acid; TG, triglyceride; TC, total cholesterol; HDL-C, high-density lipoprotein cholesterol; LDL-C, low-density lipoprotein cholesterol; MACEs, major adverse cardiovascular events; MACCEs, major adverse cardiovascular and cerebrovascular events; MI, myocardial infarction.

**Table 2:** Cox regression analysis for ACM.

Variables	B	SE	Wald	*P*	HR	95.0% CI
Gender (male)	−0.255	0.263	0.937	0.333	0.775	0.463–1.298
Age (years)	0.056	0.011	23.908	<0.001	1.057	1.034–1.081
Family history	−1.087	0.428	6.443	0.011	0.337	0.146–0.781
Diabetes mellitus	0.823	0.230	12.812	<0.001	2.278	1.451–3.576
Cr	0.006	0.001	31.504	<0.001	1.006	1.004–1.008
TG	0.023	0.034	0.456	0.500	1.023	0.958–1.092
TC	0.027	0.082	0.109	0.741	1.028	0.874–1.208
LDL-C	0.090	0.138	0.427	0.513	1.094	0.836–1.432
ALR	0.702	0.229	9.420	0.002	2.017	1.289–3.158

Cr, creatinine; TG, triglyceride; TC, total cholesterol; LDL-C, low-density lipoprotein cholesterol.

**Table 3:** Cox regression analysis for CM.

Variables	B	SE	Wald	*P*	HR	95% CI
Gender (male)	−0.861	0.388	4.922	0.027	0.423	0.197–0.905
Age (years)	0.045	0.014	9.731	0.002	1.046	1.017–1.076
Family history	−0.943	0.525	3.231	0.072	0.389	0.139–1.089
Diabetes mellitus	0.735	0.303	5.899	0.015	2.085	1.152–3.772
Cr	0.005	0.002	7.481	0.006	1.005	1.001–1.008
TG	−0.003	0.077	0.001	0.972	0.997	0.857–1.161
TC	0.080	0.103	0.596	0.440	1.083	0.884–1.326
LDL-C	0.179	0.185	0.937	0.333	1.196	0.832–1.720
ALR	0.622	0.294	4.477	0.034	1.862	1.047–3.313

Cr, creatinine; TG, triglyceride; TC, total cholesterol; LDL-C, low-density lipoprotein cholesterol.

## Data Availability

Due to confidentiality policies, data will not be shared.

## Additional Points

The limitations in our study should be detailed. First, because of only lymphocyte data and baseline ALT level collected, the dynamic changes of the two variables did not be analyzed. Second, we did not check CD4^+^ T cell level, which contributes to inflammatory response in coronary artery disease. Finally, this is a single retrospective cohort design. Results of our study need to be further verified by a multicenter, prospective study.
